# Medical Abortion of a First-Trimester Pregnancy with Large Multiple Uterine Leiomyomata

**DOI:** 10.1155/2021/9988653

**Published:** 2021-06-19

**Authors:** Somsook Santibenchakul, Unnop Jaisamrarn

**Affiliations:** ^1^Department of Obstetrics and Gynecology, King Chulalongkorn Memorial Hospital, Rama IV Road, Pathumwan, Bangkok 10330, Thailand; ^2^Department of Obstetrics and Gynecology, Faculty of Medicine, Chulalongkorn University, Rama IV Road, Pathumwan, Bangkok 10330, Thailand

## Abstract

**Introduction:**

Termination of pregnancy in a patient with huge uterine leiomyomata poses significant challenges to clinicians. In this study, we report the successful termination of pregnancy in a patient with large multiple uterine leiomyomata using a combined regimen of drugs for medical abortion.

**Case:**

A 42-year-old woman, 6 weeks pregnant, presented to the Family Planning Clinic with an unintended pregnancy. She had a large, irregular abdominal midline mass, equivalent in size to 30-32 weeks of pregnancy. Abdominal and transvaginal ultrasound examinations revealed a small intrauterine gestational sac with a yolk sac and multiple large uterine leiomyomata. Treatment with mifepristone (200 mg) was initiated at the clinic. In addition, she was instructed to sublingually take 800 *μ*g of misoprostol after 24–48 h. Two weeks later, at the follow-up visit, the patient complained of continued light bleeding. A pelvic examination showed that her cervix was dilated by 1 cm. In addition, abdominal and transvaginal ultrasound revealed a thick, inhomogeneous endometrium. Owing to light bleeding and no anemia or infection, the patient received two additional doses of 800 *μ*g misoprostol vaginally. Her bleeding subsided for 61 days, and she resumed her normal menstrual cycle.

**Conclusion:**

A first-trimester pregnancy with large multiple uterine leiomyomata can be safely terminated using a combination regimen of drugs for medical abortion. However, an additional dose of misoprostol is required for the successful termination of pregnancy.

## 1. Introduction

Termination of pregnancy in a patient with a large uterine leiomyomata poses significant challenges to clinicians. A leiomyoma itself is not considered a contraindication for either medical or surgical abortion [1–3]. However, theoretically, it reduces myometrial contractility by weakening the uterine contractile force and impairing the expulsive mechanism [4, 5]. Thus, it may decrease the success rate of medical abortions. Furthermore, a large uterine leiomyoma may distort the endometrial cavity, causing difficulties in surgical abortion [6]. Due to the rarity of this condition, only a few case reports and case series exist involving such cases [7–14] (Table 1). Therefore, this report shares our success in conservatively managing such difficult cases.

## 2. Case Summary

A 42-year-old patient who was 6 weeks pregnant, gravida 1, para 0, reported to the Family Planning Clinic due to an unintended pregnancy. The patient requested termination of pregnancy because of social and economic reasons. Her only symptom was that she had not had her menstrual period for one month. The patient's medical and surgical histories were unremarkable. Physical examination of the patient revealed a height of 154 cm, weight of 75.3 kg, and blood pressure of 110/67 mmHg. Abdominal examination revealed a large, irregular mass in the abdominal midline. During pelvic examination, the patient's vagina showed normal vaginal discharge and a closed cervix. The irregular midline mass, equivalent in size to a 30-32-week pregnancy, tended to move in conjunction with the cervix. Examination of the lower extremities revealed no signs of edema. In addition, abdominal and transvaginal ultrasound evaluation on her first visit confirmed that the patient had an intrauterine pregnancy along with multiple uterine leiomyomata. A detailed examination revealed that her uterus was 90 × 33 × 86 mm in size, including a small intrauterine gestational sac with a yolk sac. Multiple uterine leiomyomata of both intramural and subserosal types were found. These were located at the upper part of the posterior uterine wall, the upper part of the anterior uterine wall, the uterine fundus, the left lateral wall in the lower part of the uterus, and the right lateral wall in the lower part of the uterus. The sizes and volumes of these leiomyomata were (1) 35 × 20 × 32 mm (International Federation of Gynecology and Obstetrics (FIGO) classification: type O-3); (2) 12 × 13 × 10 mm (FIGO: type O-3); (3) 136 × 108 × 128 mm, 984.4 mL (FIGO: type O-6); (4) 68 × 71 × 67 mm, 169.4 mL; and (5) 94 × 65 × 94 mm, 300.7 mL, respectively. Thus, the diagnosis established based on ultrasound findings was an early intrauterine pregnancy with a large uterine leiomyomata. After comprehensive reproductive healthcare counseling, the patient decided to undergo a medical abortion.

The patient was screened for the inclusion and exclusion criteria from a clinical trial of the introduction of medical abortion in Thailand [15], which assessed the acceptance of a combined regimen of drugs for medical abortion (Medabon®; Sun Pharmaceutical Industries Ltd, Mumbai, India) for Thai women in their first-trimester pregnancy. The patient was eligible for the clinical trial; therefore, 200 mg of mifepristone was initiated at the clinic. In addition, she was instructed to take 800 *μ*g of misoprostol sublingually at home after 24–48 hours. An appointment was made to assess the completion of abortion two weeks after her first visit.

**Figure 1:**
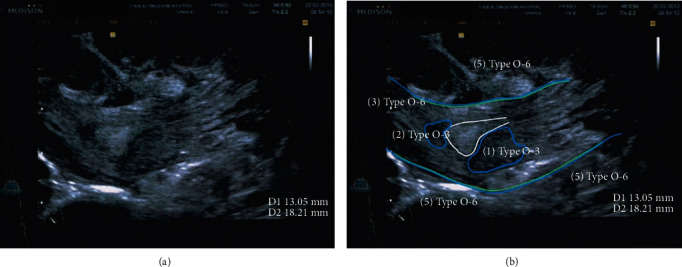
(a) Abdominal ultrasound (sagittal view of right lower abdomen) revealed an 18-millimeter-thick inhomogeneous endometrium with multiple intramural and subserosal leiomyomata (2 weeks after the combined regimen for medical abortion was administered). (b) Ultrasonic imaging revealing multiple leiomyomata (white line: endometrium; blue line: leiomyomata; green line: uterine wall) (Samsung Medison).

At the follow-up visit, the patient complained of continued light bleeding. Pelvic examination revealed that her cervix was dilated by 1 cm. In addition, abdominal and transvaginal ultrasound revealed an 18-millimeter-thick inhomogeneous endometrium (Figure 1); thus, the abortion was classified as incomplete. Owing to light bleeding and no signs of anemia or infection, the patient was counseled according to her condition and various management options. She agreed to receive two additional doses of 800 *μ*g misoprostol vaginally, which were administered 72 h apart [16]. The patient's bleeding subsided for 61 days, after which she resumed her normal menstrual cycle.

## 3. Discussion

The present case report involves a successfully managed, difficult case of conservative termination of pregnancy in a patient with large multiple leiomyomata. In our opinion, medical abortion was the preferred method of treatment in this case. An intramural leiomyoma that is located in close contact with the endometrial cavity may impede the introduction of operative instruments into the uterine cavity, resulting in technically challenging and potentially dangerous operative abortions. Therefore, a medical abortion is the preferred treatment option in such cases [6, 7]. A combined regimen of drugs for medical abortion was initiated in this case study, which comprised 200 mg of mifepristone followed by 800 *μ*g of sublingual misoprostol with a 24- to 48-hour interval. This regimen is the most effective regimen for terminating first-trimester pregnancies [17]. Mifepristone is only available in some countries, and as of 2012, this medication was not available in Thailand. Fortunately, at that time, mifepristone was used in a clinical trial that investigated the acceptability of combined regimen medical abortion in Thai women [15].

Li et al. [10] used a combination of mifepristone and misoprostol to terminate pregnancy in the participants of their study. The authors prescribed 600 mg of mifepristone followed by 600 *μ*g of misoprostol 48 h later. However, due to a lack of response to this regimen, an additional 1,000 *μ*g of misoprostol was vaginally administered. The medical abortion regimen used by Li et al. was different from our case because additional vaginal misoprostol in our case was administered because the abortion was deemed incomplete at the follow-up performed after two weeks. This case was unique in terms of the medical abortion regimen used. We followed the standard recommendations of the World Health Organization for the initial regimen for terminating the first trimester of pregnancy [18], and misoprostol was used to treat the incomplete abortion [16]. Mark et al. [8] reported a case series of 12 patients with large leiomyomata who had a successful medical abortion. For four patients who were under 14 weeks of gestation, the authors prescribed 200 mg of mifepristone followed by two doses of 800 *μ*g of vaginal misoprostol 24 and 48 hours later. This medical abortion regimen is different from the regimen recommended by the World Health Organization for termination of pregnancy in patients under 12 weeks of gestation [18]. Among these four cases, one case had an incomplete abortion with heavy bleeding requiring blood transfusion and uterine evacuation. The uterine sizes of these four cases were equivalent to those of approximately 16-20 weeks of gestation.

The major limitation of this study that must be considered before generalizing to other patients is that this procedure should only be performed with great caution. Although additional doses of misoprostol may be prescribed for patients with an incomplete abortion [16], careful evaluation of the patient for the symptoms of anemia is required. In our case, we carefully evaluated the clinical manifestations of the condition in our case before prescribing additional at-home misoprostol. The patient was instructed to return to the hospital immediately if her sanitary pads were soaked rapidly. Additionally, as this case had prolonged vaginal bleeding, concerning early signs and symptoms of any uterine infection is recommended. The patient was instructed to look for signs and symptoms of uterine infections, such as malodorous vaginal discharge, pelvic pain, and fever. She was also instructed to refrain from sexual intercourse and vaginal douching until the bleeding subsided. Lastly, as this patient had multiple uterine leiomyomata, they interfered with the quality of ultrasound imaging, and high-resolution ultrasound examination was required to evaluate both uterine leiomyomas and the completeness of abortion.

### 3.1. Patient Perspective

Our patient, who had not realized prior to visiting our clinic that she had huge leiomyomata, intended to end her pregnancy. After comprehensive counseling about the options to terminate pregnancy, she decided to choose a medical abortion to avoid a surgical procedure. The patient stated that the medicine was easy to use at home and gave her privacy. She complained of light bleeding for 61 days. This period was longer than expected. The patient also experienced abdominal discomfort, which required painkillers; however, her pain was not as severe as anticipated.

## 4. Conclusion

In this study, a first-trimester pregnancy with large multiple uterine leiomyomata could safely be terminated by using a combination regimen of medical abortion, comprising 200 mg of mifepristone followed by 800 *μ*g of sublingual misoprostol with a 24- to 48-hour interval. However, an additional dose of misoprostol was still required, as the patient experienced continued bleeding due to an incomplete abortion. Clinicians must pay extra attention to any signs and symptoms of both anemia and uterine infection. A high-resolution ultrasound was useful in evaluating the uterine leiomyomata and the success of the abortion.

## Figures and Tables

**Table 1:** List of a case series and seven reported cases of patients with large uterine leiomyomata who underwent abortion.

Authors/year of publication	Gestational age (weeks)/remarks	Description of leiomyoma/size of uterus	Description of intervention	Induction to abortion time
Saad-Naguib et al. [7] 2017	9	18.1 × 9.0 × 15.7 cm of subserosal leiomyoma at posterior wall of uterus 20-week pregnancy	1 mEq/mL of fetal intracardiac potassium chloride injection followed by 2 doses of 20 mg/m^2^ methotrexate	2 weeks
Mark et al. [8] 2016	10-20 Case series of 12 patients	Uterine sizes range from 16- to 32-week pregnancy	For patients up to 14-week pregnancy, 200 mg of oral mifepristone followed by 2 doses of 800 *μ*g misoprostol vaginally 24 and 48 h For patients beyond 14-week pregnancy, 200 mg of oral mifepristone followed by 400 *μ*g of misoprostol vaginally every 4–6 h until delivery	6-17 h 3 patients underwent uterine evacuation due to incomplete abortion
Seto et al. [9] 2013	14 Prior classical cesarean section	14 × 10 × 9 cm subserous leiomyoma arising from posterior aspect of uterus 16-18-week pregnancy	Dilapan®-S^1^ intracervically 12 h before 3 doses of misoprostol vaginally every 4 h (50, 100, and 150 *μ*g, respectively)	9.5 h after first dose of misoprostol
Li et al. [10] 2005	6	Nine measurable leiomyomata, the largest of which measured 10 × 9 × 9 cm and was located in the posterior lower uterine segment 24-week pregnancy	3 tablets of 200 mg mifepristone and 3 tablets of 200 *μ*g misoprostol 48 h later and then 5 tablets of 200 *μ*g misoprostol vaginally	NA^2^
Dalton and Lebovic [11] 2003	11 3 prior unsuccessful evacuation attempts	Multiglobular, heterogenous masses with the largest's diameter measuring 8 cm in the posterior corpus, extending cephalad, and caudad NA^2^	Surgical abortion using a flexible cannula	NA^2^
Fenwick and Divers [12] 1995	6	14 × 10 × 12 cm intramural leiomyoma 24-week pregnancy	600 mg mifepristone orally, followed by 1 mg gemeprost vaginally after 36 h	NA^2^
Creinin [13] 1996	6-7 History of myomectomies	Multiple leiomyomata 25-week pregnancy	104 mg of methotrexate intramuscularly, followed by four tablets of 200 *μ*g misoprostol vaginally 6 days later	3 h after first dose of misoprostol
Buckshee and Dhond [14] 1992	10	Large multiple leiomyomata 32-34-week pregnancy	Intra-amniotic and intraplacental instillation of methotrexate, 25 mg at each site	8 days

^1^Dilapan®-S (synthetic osmotic cervical dilator; Gel-Med International, Kamenne Zehrovice, Czech Republic). ^2^No information available.
